# Germline mismatch repair (MMR) gene analyses from English NHS regional molecular genomics laboratories 1996–2020: development of a national resource of patient-level genomics laboratory records

**DOI:** 10.1136/jmg-2022-108800

**Published:** 2022-12-26

**Authors:** Lucy Loong, Catherine Huntley, Fiona McRonald, Francesco Santaniello, Joanna Pethick, Bethany Torr, Sophie Allen, Oliver Tulloch, Shilpi Goel, Brian Shand, Tameera Rahman, Margreet Luchtenborg, Alice Garrett, Richard Barber, Tina Bedenham, David Bourn, Kirsty Bradshaw, Claire Brooks, Jonathan Bruty, George J Burghel, Samantha Butler, Chris Buxton, Alison Callaway, Jonathan Callaway, James Drummond, Miranda Durkie, Joanne Field, Lucy Jenkins, Terri P McVeigh, Roger Mountford, Rodney Nyanhete, Evgenia Petrides, Rachel Robinson, Tracy Scott, Victoria Stinton, James Tellez, Andrew J Wallace, Laura Yarram-Smith, Kate Sahan, Nina Hallowell, Diana M Eccles, Paul Pharoah, Marc Tischkowitz, Antonis C Antoniou, D Gareth Evans, Fiona Lalloo, Gail Norbury, Eva Morris, John Burn, Steven Hardy, Clare Turnbull

**Affiliations:** 1 Division of Genetics and Epidemiology, The Institute of Cancer Research, Sutton, UK; 2 NHS Digital, National Disease Registration Service, London, UK; 3 Health Data Insight CIC, Cambridge, UK; 4 Centre for Cancer, Society & Public Health, King's College London, London, UK; 5 Central and South Genomic Laboratory Hub, West Midlands Regional Genetics Laboratory, Birmingham, UK; 6 West Midlands, Oxford and Wessex Genomic Laboratory Hub, Oxford University Hospitals NHS Foundation Trust, Oxford, UK; 7 North East and Yorkshire Genomic Laboratory Hub, Newcastle Upon Tyne Hospitals NHS Foundation Trust, Newcastle Upon Tyne, UK; 8 East Midlands and East of England Genomics Laboratory, Nottingham University Hospitals NHS Trust, Nottingham, UK; 9 North Thames Genomic Laboratory Hub, Great Ormond Street Hospital for Children NHS Foundation Trust, London, UK; 10 East Genomic Laboratory Hub, Cambridge University Hospitals Genomic Laboratory, Cambridge University Hospitals NHS Foundation Trust, Cambridge, UK; 11 Manchester Centre for Genomic Medicine and North West Genomic Laboratory Hub, Manchester University NHS Foundation Trust, Manchester, UK; 12 Bristol Genetics Laboratory, Southmead Hospital, Bristol, UK; 13 Wessex Regional Genetics Laboratory, Salisbury Hospital NHS Foundation Trust, Salisbury, UK; 14 Sheffield Diagnostic Genetics Service, North East and Yorkshire Genomic Laboratory Hub, Sheffield Children's NHS Foundation Trust, Sheffield, UK; 15 Cancer Genetics Unit, Royal Marsden Hospital NHS Trust, London, UK; 16 North West Genomic Laboratory Hub (Liverpool), Manchester Centre for Genomic Medicine, Liverpool, UK; 17 Yorkshire and North East Genomic Laboratory Hub, Leeds Teaching Hospitals NHS Trust, Leeds, UK; 18 The Ethox Centre and Wellcome Centre for Ethics and Humanities, Nuffield Department of Population Health, University of Oxford Ethox Centre, Oxford, UK; 19 Cancer Sciences, University of Southampton Faculty of Medicine, Southampton, UK; 20 Human Genetics and Genomic Medicine, Faculty of Medicine, University of Southampton, Southampton, UK; 21 Department of Medical Genetics, NIHR Cambridge Biomedical Research Centre, Cambridge, UK; 22 Division of Evolution & Genomic Sciences, The University of Manchester, Manchester, UK; 23 South East Genomic Laboratory Hub, Guy's and St Thomas' Hospitals NHS Trust, London, UK; 24 Nuffield Department of Population Health, University of Oxford, Oxford, UK; 25 Translational and Clinical Research Institute, Newcastle University, Newcastle upon Tyne, UK

**Keywords:** Genomics, Databases, Genetic, Genetics, Medical, Genetics, Population, Genetic Testing

## Abstract

**Objective:**

To describe national patterns of National Health Service (NHS) analysis of mismatch repair (MMR) genes in England using individual-level data submitted to the National Disease Registration Service (NDRS) by the NHS regional molecular genetics laboratories.

**Design:**

Laboratories submitted individual-level patient data to NDRS against a prescribed data model, including (1) patient identifiers, (2) test episode data, (3) per-gene results and (4) detected sequence variants. Individualised per-laboratory algorithms were designed and applied in NDRS to extract and map the data to the common data model. Laboratory-level MMR activity audit data from the Clinical Molecular Genetics Society/Association of Clinical Genomic Science were used to assess early years’ missing data.

**Results:**

Individual-level data from patients undergoing NHS MMR germline genetic testing were submitted from all 13 English laboratories performing MMR analyses, comprising in total 16 722 patients (9649 full-gene, 7073 targeted), with the earliest submission from 2000. The NDRS dataset is estimated to comprise >60% of NHS MMR analyses performed since inception of NHS MMR analysis, with complete national data for full-gene analyses for 2016 onwards. Out of 9649 full-gene tests, 2724 had an abnormal result, approximately 70% of which were (likely) pathogenic. Data linkage to the National Cancer Registry demonstrated colorectal cancer was the most frequent cancer type in which full-gene analysis was performed.

**Conclusion:**

The NDRS MMR dataset is a unique national pan-laboratory amalgamation of individual-level clinical and genomic patient data with pseudonymised identifiers enabling linkage to other national datasets. This growing resource will enable longitudinal research and can form the basis of a live national genomic disease registry.

**WHAT IS ALREADY KNOWN ON THIS TOPIC**

- Several studies have reported (1) the frequency of germline mismatch repair (MMR) gene analyses in institution-specific cancer cohorts and (2) retrospectively and prospectively observed cancer incidence for MMR mutation carriers.
- There are no amalgamated national data detailing the frequency and patterns of MMR analyses.

**WHAT THIS STUDY ADDS**

- This study provides the first detailed population-based national overview of the totality of germline MMR gene analyses conducted within the English NHS, with pseudonymised, individual-level data available for >60% of patients tested.
- This amalgamation of individual patient-level laboratory data for both normal and abnormal results from MMR gene testing enables detailed examination of the patterns of gene testing, abnormal results, variants detected and via record linkage to the English national cancer registry, cancers arising in individuals who received MMR testing.

**HOW THIS STUDY MIGHT AFFECT RESEARCH, PRACTICE OR POLICY**

- We report a new national patient-level laboratory data collection from all NHS regional molecular genomics laboratories in England, which will be a growing, dynamic resource housed within the National Disease Registration Service.
- Currently, this dataset captures >60% of NHS germline MMR gene analyses performed in England to date, including all of the MMR full-gene analyses since 2016. This resource provides unique opportunities for patient-level record linkage of germline MMR genetic data to nationally collected cancer registrations, treatment and outcomes, thus providing infrastructure by which to initiate a national Lynch Syndrome registry.
- This study illustrates the wide national variability in local laboratory informatic systems by which patient and genomic data are processed and stored. Going forward, coordinated national focus on laboratory data systems is urgently required if we are to optimise high-quality national amalgamation of genomic and clinical data.

## Introduction

Lynch syndrome (LS) is a hereditary cancer predisposition syndrome caused by pathogenic germline genetic variants in one of four mismatch repair (MMR) genes, *MSH2*, *MLH1*, *PMS2* and *MSH6*.^1^ It is associated with elevated risk of colorectal, endometrial, ovarian, upper urinary tract, upper gastrointestinal tract, brain and prostate cancers.^2^ Management of LS includes early-onset endoscopic surveillance of the gastrointestinal tract, aspirin chemoprophylaxis and risk-reducing gynaecological surgery. Diagnosis of LS may also influence management of cancers when they arise. Approximately 1 out of 300 of the general population and 1 out of 30 of those presenting with either colorectal or endometrial cancer have an underlying diagnosis of LS, making it one of the most common genetic cancer susceptibility syndromes.^3–5^

Prior to 2017, most testing for LS in England occurred in clinical genetics and was focused on individuals preselected on the basis of their personal and family history of cancer.^6 7^ Such testing now runs alongside universal screening of prospectively identified bowel and endometrial cancers, as recommended by the National Institute for Health and Care Excellence (NICE) in 2017 and 2020, respectively.^8–10^ Diagnostic testing for LS is typically performed in two steps.^11^ First, tumour tissue is examined for the molecular MMR phenotype of microsatellite instability (MSI) or for evidence of deficiency of MMR proteins via immunohistochemistry (IHC). If tumour tissue analysis is abnormal or the pattern of cancers in the family is highly suggestive of LS, the full genetic sequence of the MMR genes is analysed for ‘germline abnormalities’ in a constitutional sample, typically blood (full-gene analysis). For each genetic variant identified on full-gene analysis, a range of evidence is assessed by clinical scientists to assign whether the variant is pathogenic (P), likely pathogenic (LP), uncertain (VUS), likely benign (LB) or benign (B). Following identification of a P or LP variant (hereafter referred to collectively as pathogenic variant (PV)) in one family member, targeted analysis for that specific variant is offered to relatives.

In the National Health Service (NHS) of England, germline genetic testing has been provided by a network of 18 regional molecular genetics laboratories. Since initiation of NHS germline MMR gene analyses (hereafter referred to as NHS MMR analyses) in 1996, 13 out of 18 laboratories have delivered NHS MMR analyses for some/all of the period 1996–2020. Until recently, all details regarding these analyses were held separately on local laboratory systems.

The National Disease Registration Service (NDRS), part of NHS Digital and formerly part of Public Health England (until 2021), comprises the National Cancer Registration and Analysis Service (NCRAS) and the National Congenital Anomaly and Rare Disease Registration Service (NCARDRS).^12 13^ NDRS is responsible for the collection, curation, quality assurance and analysis of data relating to individuals with rare disease and/or cancer in England. In addition to the basic cancer registration record, NCRAS holds datasets on chemotherapy (Systemic Anti-Cancer Therapy Dataset, SACT), radiotherapy (National Radiotherapy Dataset, RTDS), hospital episodes (Hospital Episode Statistics, HES) and recently added a dataset of genetic alterations in cancers (somatic alterations).^14–16^ NDRS provides, therefore, an opportunity to link between datasets.

There has been increasing focus on the importance of national and international amalgamation of patients’ genomic data, reflected in initiation of bodies such as the Global Alliance for Genomics and Health (GA4GH).^17^ An amalgamated dataset of NHS germline cancer susceptibility gene analyses linked to a national cancer registry has numerous potential applications for germline variant interpretation, evaluation of national testing pathways, studying outcomes for patients with genetic predisposition to cancer and the creation of patient registries.

Here, we describe a national programme of amalgamation into the NDRS of pseudonymised patient-level laboratory data from NHS MMR analyses dating back to 2000, from the NHS regional molecular genetics laboratories of England. We describe challenges in genetic data amalgamation and analyse historic patterns and volumes of testing to inform discussions on national strategies for Lynch testing.

## Material and methods

### Pseudonymisation, data extraction and restructuring of laboratory data submissions

In 2016, the 18 NHS regional molecular genetics laboratories of England were surveyed regarding whether they currently or previously performed NHS MMR analyses. Laboratories who reported having performed NHS MMR analyses were supplied with the NDRS common data model to support design of data extracts from their Laboratory Information Management Systems (LIMS). No constraints were imposed regarding format of submitted data extracts. Following iterative optimisation and testing by NDRS of sample data extracts supplied by each laboratory, finalised extracts were submitted via a dedicated application programming interface (API). Historic data dating back as far as locally feasible were incorporated in the first submission, followed by regular submissions of prospective data. Prior to 1 October 2021, data were collected in the NDRS under the legal permissions afforded by Section 251 of the Health and Social Care Act 2006 and subsequently under Section 254 of the Health and Social Care Act 2012.^18 19^

**Figure 1:**
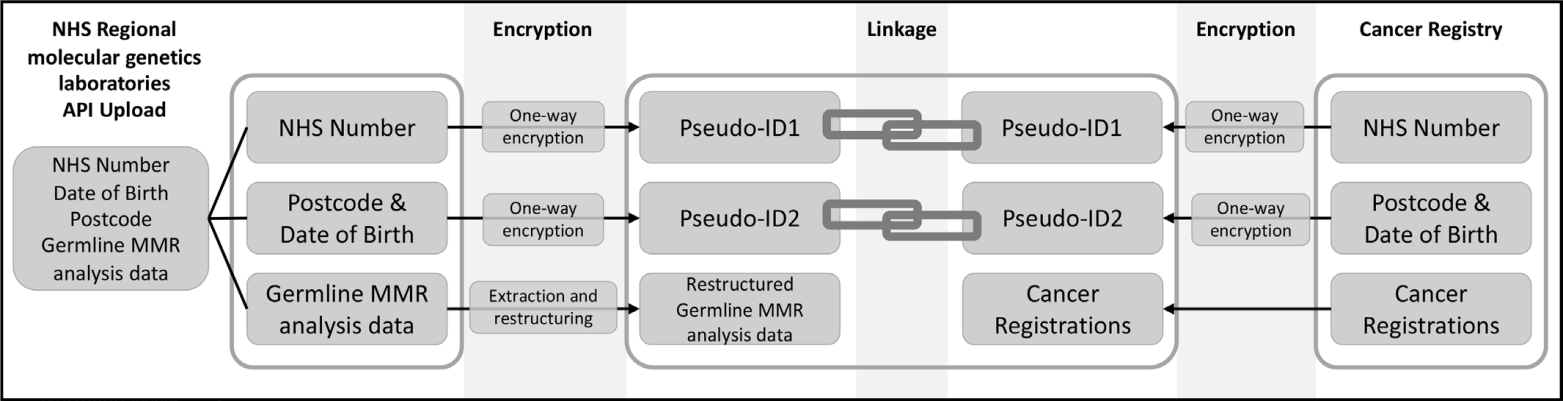
Schematic showing encryption of NHS numbers to form pseudo-ID1 and dates of birth and postcode combinations into pseudo-ID2 at the point of upload for patient-level records in the laboratory data extracts. The pseudo-IDs are recreated from NHS numbers, dates of birth and postcodes held in the cancer registry to facilitate linkage of records. API, application programming interface; MMR, mismatch repair; NHS, National Health Service.

To facilitate eventual linkage of the data to cancer registrations and other NDRS datasets, reproducible encrypted pseudonyms were created for each patient-level record in the submitted data extracts on upload. Pseudo-ID1 was created from the NHS number and pseudo-ID2 from the postcode and date of birth (DOB). Patient identifiers were automatically removed by the API and not received by NDRS. Pseudo-ID1 and pseudo-ID2 were recreated from NHS numbers, postcodes and DOBs held in the cancer registry and matching on the pseudo-IDs was then performed (figure 1). NDRS have undertaken separate validation of the pseudonymisation and linkage processes (see online supplemental methods for details and results).

Due to variations between laboratories in the structure of the submitted data extracts and field contents, bespoke algorithms were created to map the submitted data to the NDRS common data model and to derive required data items. Many laboratories submitted variant data embedded in free text (as per clinical report wording), requiring algorithms to recognise and extract Human Genome Variation Society (HGVS)-compliant variant nomenclature. Data items in the NDRS common data model included (1) pseudonymised patient identifiers, (2) test episode data, (3) per-gene results and (4) detected sequence variants (online supplemental table 1). Each laboratory-specific algorithm was iterated and optimised until the total number of tests and abnormal results for each gene was ≥95% concordant between computational and manual extractions of the originally submitted data extracts (online supplemental methods).

10.1136/jmg-2022-108800.supp3 Supplementary data

### Imputation of total historic national laboratory activity

The number of NHS MMR analyses undertaken at each laboratory (total, full-gene and targeted) pre-dating the earliest submissions from each laboratory to NDRS was estimated. This allowed estimation of overall numbers of NHS MMR analyses conducted since initiation of this testing in 1996 and the proportion of analyses captured in the NDRS germline MMR dataset. For this purpose, data were retrieved from the Clinical Molecular Genetics Society/Association of Clinical Genomic Science (CMGS/ACGS) annual per-laboratory audit of MMR analyses, covering financial years 1998–2016. These CMGS/ACGS counts included all English NHS MMR analyses (full-gene and targeted) performed by each laboratory per financial year, but for some laboratories were inflated by inclusion of tests for other patients (devolved nations, overseas, private, research) and MSI analyses. Data comparison for the years where NDRS and CMGS/ACGS data overlapped enabled centre-specific down-adjustment of the MMR analyses counts in the CMGS/ACGS audit data, to account for the inflation of these counts by non-English/non-NHS/MSI analyses, and thus approximate the numbers of full-gene and targeted tests (online supplemental methods and online supplemental table 2).

10.1136/jmg-2022-108800.supp1 Supplementary data

Estimated counts of total, full-gene and targeted NHS MMR analyses in the entire period between financial years April 1996 and March 2020 were derived from combination of counts of NHS MMR analyses in the NDRS germline MMR dataset with the down-adjusted counts derived from CMGS/ACGS audit data for the years pre-dating NDRS data submission.

### Analysis and data linkage

Descriptive analysis of the NDRS germline MMR dataset was limited to tests authorised between calendar years 2001 and 2019 inclusive. Descriptive analyses included historic patterns, volumes and results of MMR gene testing and linkage of the NDRS germline MMR dataset to the NCRAS cancer registry. NDRS data extraction and restructuring were incomplete at the time of analysis for Liverpool Genetics Laboratory (full-gene and targeted tests, n=479) and Sheffield Diagnostic Genetics Service (targeted tests only, n=146); these data comprising 625 out of 16 722 patients (3.7% of NHS MMR analyses) were not included in subsequent descriptive analyses.

Where multiple MMR analyses with different test authorisation dates existed for a single patient, these were collapsed into test episodes of maximum 365 days, with the earliest authorisation date taken as the test episode date. Patient-level records in the NDRS germline MMR dataset were deduplicated using matching pseudo-IDs and test episode dates. Linkage to NDRS cancer registrations was undertaken using pseudo-ID1 and pseudo-ID2. When linkage was successful, ICD-10 cancer site codes were retrieved (online supplemental methods).

Due to variation in the earliest patient-level data that laboratories were able to submit and non-inclusion of data from Liverpool Genetics Laboratory (which conducted NHS MMR analyses between 2004 and 2016), the descriptive analyses only reflect all active laboratories for full-gene tests from 2017 onwards. For earlier dates, the full-gene analyses reflect activity in a subset of laboratories.

### Patient and public involvement

NDRS is committed to extensive Patient and Public Involvement (PPI), including running public awareness campaigns, webinars, providing publicly downloadable reports and opportunities for public consultation and representation.^20–22^ Additionally, within the Cancer Research UK (CRUK)-funded CanGene-CanVar initiative, on 21 June 2021, Ethox investigators Hallowell and Sahan undertook a 2-hour consultation with seven members of the CanGene-CanVar patient reference panel, which included ethical considerations relating to the routine registration of patient cancer data in the NDRS repository.

### Funding

Funding for data collection and analyses has been provided by CRUK Catalyst Award CanGene-CanVar (C61296/A27223) and Bowel Cancer UK (18PG0019).

## Results

### Coverage of MMR data by time and geography

Out of 18 regional molecular genetics laboratories, 13 reported having performed NHS MMR analyses. All 13 of these laboratories submitted data to NDRS. All 13 laboratories submitted full-gene MMR analyses. Only 9 out of 13 laboratories were able to submit data on targeted tests.

The NDRS germline MMR dataset (accessed 6 November 2022) included patient-level data from 16 722 patients who had received NHS MMR analyses (9649 full-gene and 7073 targeted) with submissions dating through to November 2021. None of the 13 laboratories were able to extract data for all of their historic MMR analyses, and the earliest patient-level data submitted to NDRS ranged from 2000 to 2015 (online supplemental figure 1).

10.1136/jmg-2022-108800.supp2 Supplementary data

**Figure 2:**
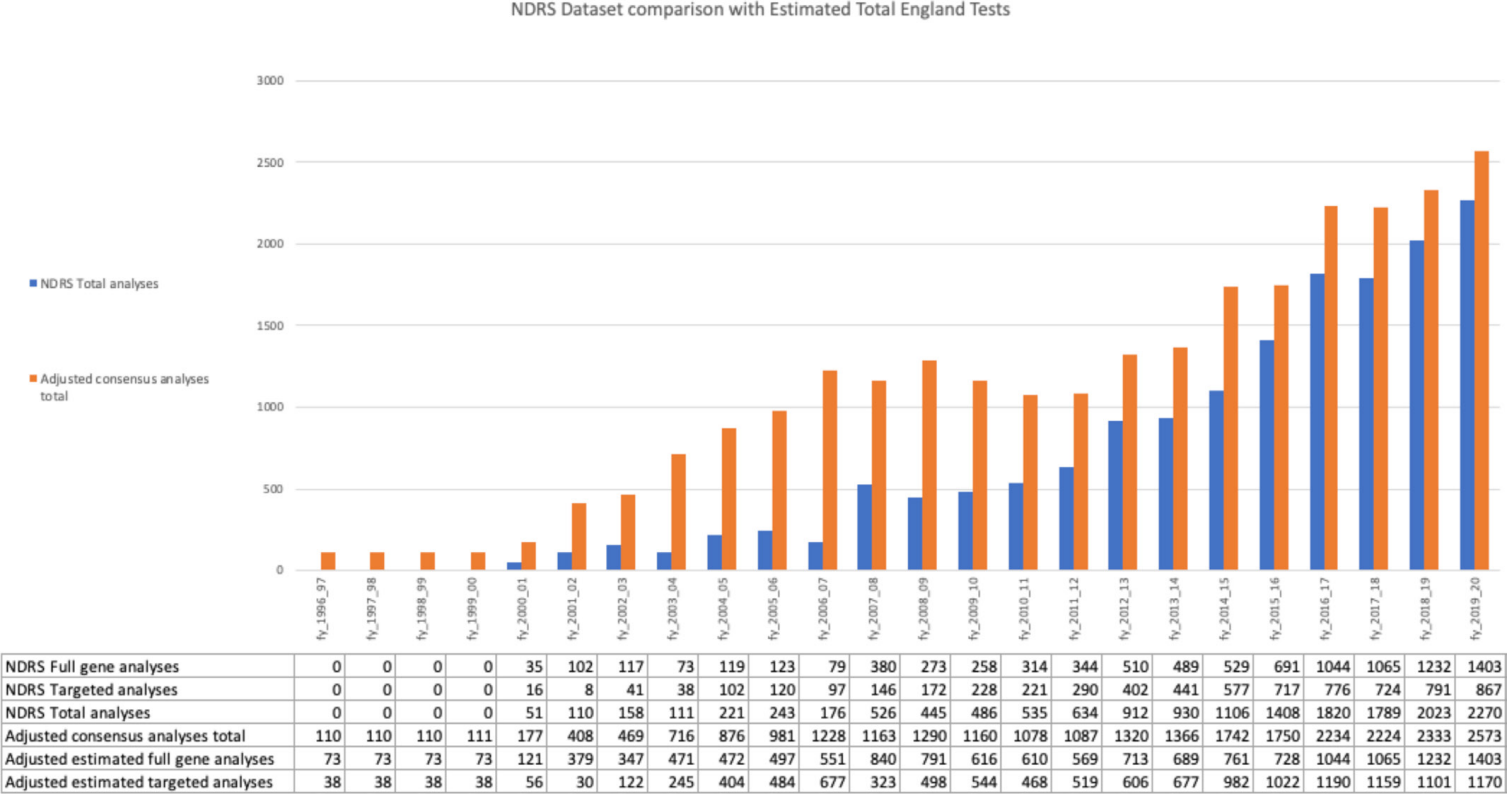
NDRS germline MMR dataset comparison with estimated total NHS germline MMR gene analyses in England for financial years April 1996–March 2020. Y-axis: number of patients who received an NHS MMR analysis. X-axis: financial year (fy). Blue bars: NDRS total tests−total number of patients who received an NHS MMR analysis captured in the NDRS germline MMR dataset. Orange bars: Adjusted consensus analyses total−estimate of the national total NHS MMR analyses undertaken, calculated from integration of NDRS and CMGS/ACGS audit data. The table beneath the X-axis shows the NDRS germline MMR dataset and adjusted consensus analyses totals broken down into full gene and targeted analyses. (See online supplemental methods and online supplemental table 2 for calculation.) Both NDRS and CMGS/ACGS data included a small number of repeat MMR analyses for patients returning to clinical genetics services and receiving subsequent MMR gene analyses. Patients in the NDRS germline MMR dataset with >1 test episode=439 (see online supplemental methods). CMGS/ACGS, Clinical Molecular Genetics Society/Association of Clinical Genomic Science; MMR, mismatch repair; NDRS, National Disease Registration Service; NHS, National Health Service.

By integrating counts of NHS MMR analyses in the NDRS germline MMR dataset through March 2020 with down-adjusted estimates of MMR analyses counts from CMGS/ACGS audit data for financial years 1998–2015 (see the Methods section), we estimated a total of 26 398 NHS MMR analyses were conducted in England from financial years Apr 1998–Mar 2020. Including interpolation of activity for the 2 years of NHS MMR analysis activity that predate CMGS/ACGS audit data (1996–1998), this estimate increased to 26 619, comprising 14 191 full-gene and 12 428 targeted analyses. Per-laboratory national and temporal coverage from integration of NDRS and CMGS/ACGS analyses counts is shown in figure 2, online supplemental table 2 and online supplemental figure 1. Overall, the NDRS germline MMR dataset is estimated to capture the individual patient-level data of ~60% of the estimated total NHS MMR analyses from first delivery of NHS MMR analyses in 1996 until censoring of NDRS MMR data at March 2020.

There was nationally complete patient-level data from all 13 laboratories for full-gene NHS MMR analyses for the four financial year (fy_) period of April 2016–Mar 2020 during which 4744 patients underwent full-gene testing for one or more MMR genes. This represents a mean annual national rate for England of MMR analyses during that period of 1186 full-gene analyses/year. Overall, the number of patients in England undergoing NHS MMR analyses has exhibited a steady increase over time (figure 2). Between April 2016–Mar 2020, the number of full-gene NHS MMR analyses increased from 1044 in fy_2016, to 1065 in fy_2017, to 1232 in fy_2018, to 1403 in fy_2019, a 34% increase from fy_2016 to fy_2019 (online supplemental table 2).

The following descriptive analyses were conducted on the NDRS germline MMR dataset limited to NHS MMR analyses authorised between calendar years 2001 and 2019 inclusive, incorporating data from 12 out of 13 laboratories (see the Methods section). These analyses comprise data on 14 583 patients, of whom 8373 underwent full-gene analyses and 6210 targeted analyses.

### Pattern of gene testing

**Figure 3:**
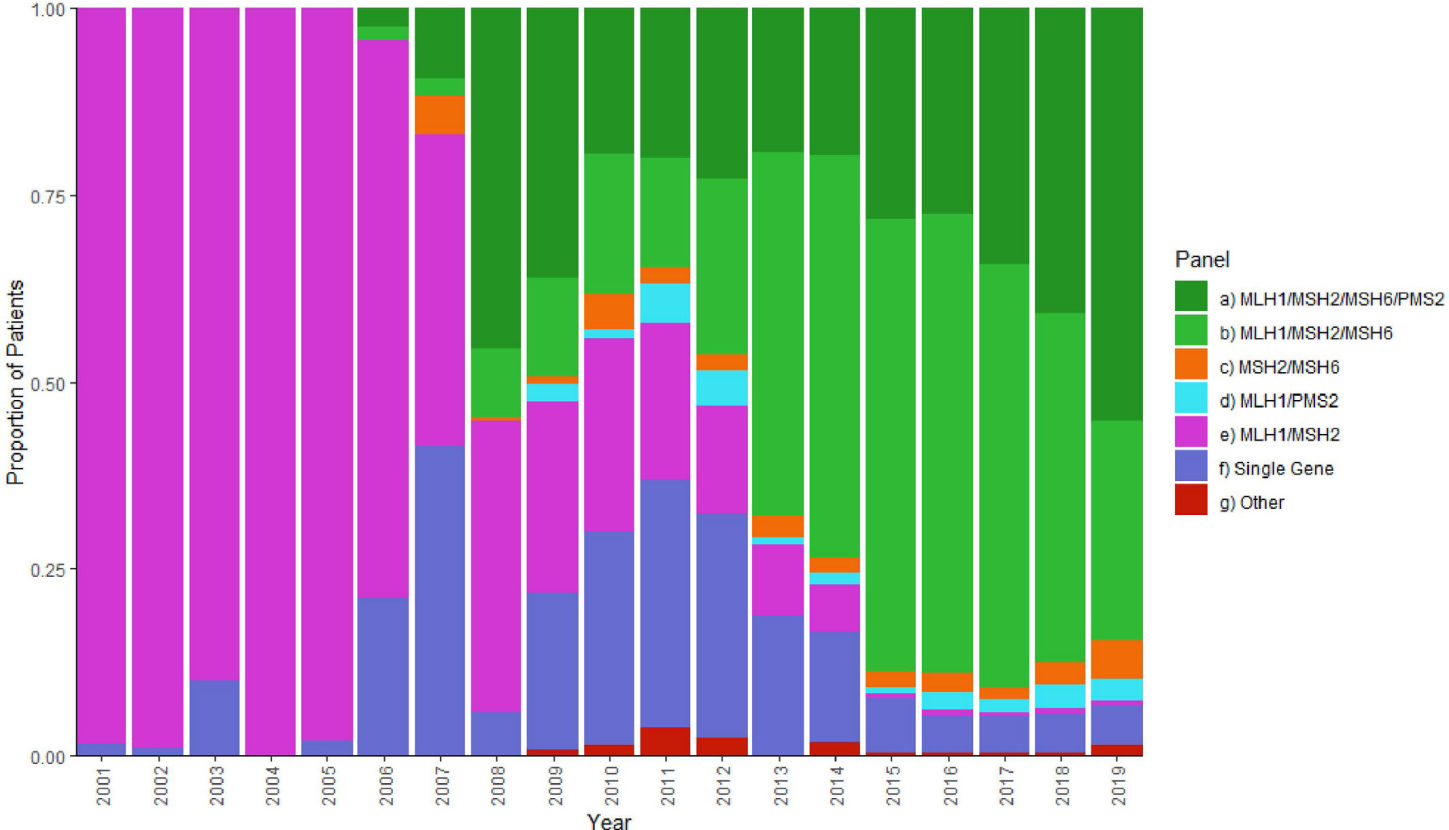
Combinations of MMR genes analysed together over time in the NDRS germline MMR dataset 2001–2019. Only full-gene analyses in patients’ first test episodes are included to represent the testing patients received on their first contact with a clinical genetics service in a given year. X-axis: calendar years. Y-axis: proportion of patients in a given calendar year receiving each combination of gene analyses. MMR, mismatch repair; NDRS, National Disease Registration Service.

Until 2008, the predominant pattern of full-gene analysis was *MLH1*/*MSH2* in combination or as single genes. Analysis of *PMS2* and *MSH6* was first reported in 2006 with testing of three/four genes being offered increasingly commonly from 2008 (figure 3, online supplemental figure 2). These patterns potentially reflect the later discovery of the ‘newer’ MMR genes *MSH6* and *PMS2*, changes in MMR tumour ‘screening’ practices from MSI to IHC, difficulties establishing assays for *PMS2* due to the presence of pseudogenes, and more recently increased capacity of Next-generation Sequencing (NGS) to deliver panel testing.

### Data linkage and cancer status of patients tested

For 90.2% (13 150/14 583) of patients in the NDRS germline MMR dataset, both pseudo-ID1 and pseudo-ID2 were available, maximising potential for successful, accurate data linkage. For 0.5% (72/14 583), only pseudo-ID1 was available; for 8.6% (1,256/14,583), only pseudo-ID2 was available; and for 0.7% (105/14,583), no linkage pseudonyms could be created. This proportion varied over time with >95% of patient-level records after 2011 having both pseudo-ID1 and pseudo-ID2 available. Due to the timing of adoption of NHS numbers, prior to 2008, most patient-level records had only pseudo-ID2 available (figure 1,online supplemental methods, online supplemental figure 3).

**Figure 4:**
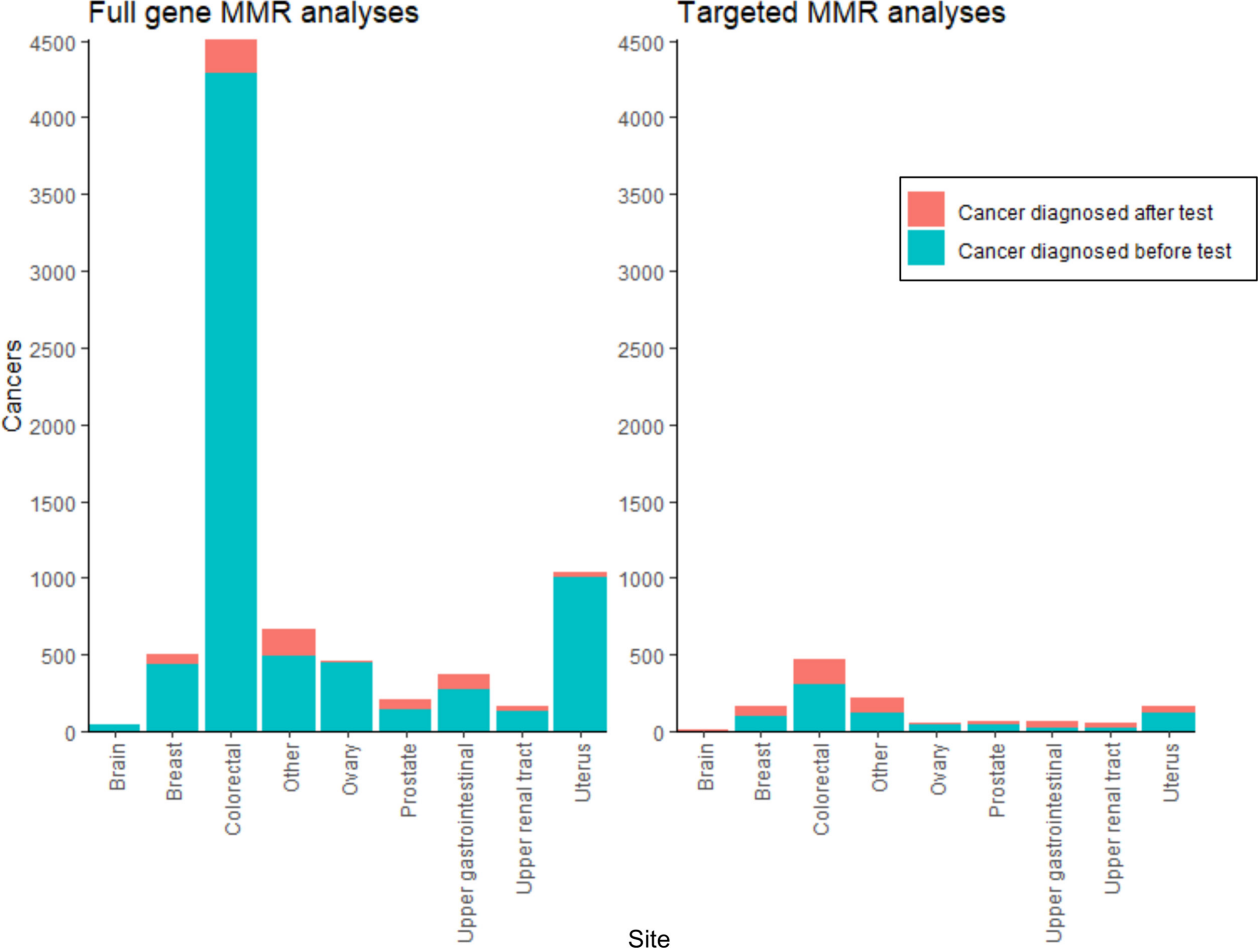
Cancer registrations linked to the NDRS germline MMR dataset. X-axis: cancer site; Y-axis: number of unique cancers registered in the NDRS national cancer registry diagnosed between 1995 and 2019 inclusive (multiple primaries, multiple cancer sites and cancers diagnosed before and after a genetic test in a single patient have all been included). Turquoise: cancers diagnosed before the genetic test report date. Orange: cancers diagnosed after the genetic test report date (for patients with multiple test episodes, this is relative to the first test episode for that patient). Plot separated into cancers linked to full-gene MMR analyses (left) and targeted MMR analyses (right). MMR, mismatch repair; NDRS, National Disease Registration Service.

Via linkage to the National Cancer Registry using pseudo-ID1 and pseudo-ID2, 70% (5831/8282) of patients who had full-gene MMR analysis and had linkage pseudonyms available, had one or more pretest diagnoses of cancer. These pretest cancers comprised: 4289 colorectal cancers (ICD10 C18-20), 1003 uterine cancers (ICD10 C54-55) and 1947 other cancers (1145 patients had >1 pretest diagnosis of cancer). About 15% (946/6196) of patients who had targeted MMR analysis and had linkage pseudonyms available, had a registered cancer. In 646 patients, the diagnosis was prior to, and in 408 patients, the diagnosis was subsequent to germline testing (108 patients had a cancer diagnosed both before and after the test) (figure 4, online supplemental table 3). For cancer probands in whom germline MMR testing was performed subsequent to their cancer diagnosis, the median age at diagnosis was 51 for colorectal cancer and 54 for endometrial (online supplemental table 4).

### Identification of abnormal variants

Normal patient-level results are defined as those (1) labelled by the submitting laboratory as ‘negative results’ and/or (2) containing only variant(s) classified by the laboratory as B or LB. Abnormal results are defined as those (1) labelled by the submitting laboratory as ‘positive results’ and/or (2) contain rare variants which are labelled by the laboratory as VUS, LP, P or unclassified. For patients with multiple variants potentially in multiple genes, only the most significant result (P>LP>VUS>abnormal unclassified>normal) for that patient test episode is counted.

**Figure 5:**
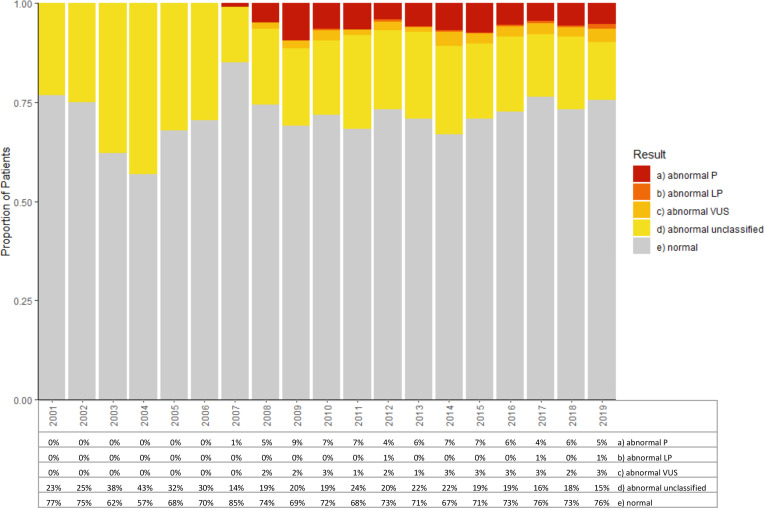
Result breakdown and availability of pathogenicity classification for full-gene germline MMR analyses by year. For patients found to have multiple variants potentially in multiple genes, only the most significant result (P>LP>VUS>abnormal unclassified>normal) for that patient test episode is counted. For the small number of patients with multiple full-gene test episodes in different years, the result of each test episode is represented in the respective year. Abnormal unclassified refers to results labelled by the submitting laboratory as abnormal but for which no pathogenicity classification was available. X-axis: calendar years; Y-axis: proportion of patients tested that year; Table: percentage of patients tested that year with a given result outcome. MMR, mismatch repair; LP, likely pathogenic; P, pathogenic; VUS, variant of uncertain significance.

The proportion of the 8373 full-gene analyses with an abnormal result is roughly consistent from 2008 onwards at ~28% (figure 5). Of the data submissions corresponding to these abnormal results, a variant could be computationally extracted in 76% of cases, of which 96% were correct on basic HGVS nomenclature checking (Mutalyzer V.2.0.35, online supplemental methods). Of the 24% of abnormal results where a variant was not computationally extracted, inspection of the raw data submissions revealed that >80% were copy number variants described using highly variable natural language terminologies rather than HGVS-compliant variant nomenclature. Overall, variant pathogenicity classifications were only provided by the submitting laboratory for 29% of abnormal results (figure 5). For the 4 out of 12 laboratories that provided pathogenicity classifications for all abnormal results including copy number variants, the rate of identification of a P or LP variant among unique patients undergoing full-gene MMR analyses was 15% (14% P, 1% LP, 6% VUS, 79% LB/B/No variant), with 71% of abnormal results being P/LP (online supplemental table 5).

## Discussion

We have presented an overview of the patient-level NHS MMR analyses amalgamated within the NDRS germline MMR dataset. These data provide opportunity for detailed analyses of the volumes of gene testing, patterns of genes analysed, frequency of abnormal results and, via linkage within NDRS to cancer registrations, of the pre-genetic and post-genetic test cancer profiles of patients undergoing NHS MMR analyses. We have estimated the historic completeness of the NDRS germline MMR dataset using CMGS/ACGS laboratory-level audit data dating back to 1998. Combined, these data provide comprehensive insights into NHS MMR analyses performed across all English NHS regional molecular genetic laboratories since initiation of NHS MMR analyses in 1996.

With patient-level data from 16 722 patients (accessed 11 June 2022) the NDRS germline MMR dataset is the largest single-country dataset of germline MMR testing reported to date. Storage within NDRS means this dataset can be linked to pre-existing national datasets of cancer registrations, treatments and outcomes and therefore can form the basis for a prospective cohort study of individuals who have undergone MMR testing, to answer key questions regarding the diagnosis and management of patients with LS. Additionally, as the NDRS germline MMR dataset captures normal MMR analyses too, there are numerators and denominators for variants observed, which are useful for interpretation of VUSs.^23^

These opportunities will compliment those afforded by the Prospective Lynch Syndrome Database which comprises 5199 MMR mutation carriers, ascertained across multiple countries with different LS diagnostic pathways and management protocols.^2^

### National NHS MMR testing activity

Although derived for earlier years from approximations based on audit data, the low volume of MMR analyses undertaken in England between April 1996 and March 2020 is striking. The NDRS germline MMR dataset is nationally complete for full-gene MMR analyses conducted between April 2016 and March 2020. Even during this period when NGS technologies are well established, on average, only 1186 patients received a full-gene MMR analysis per year in England, a country with population of ~56 million. In total for the period April 1996–March 2020, we estimate full-gene analyses were performed on 14 191 patients and targeted analyses on 12 428 patients. The rate of detection of P/LP variants in full-gene analyses where provided in the NDRS data was on average 15% (online supplemental table 5) and the rate of abnormal results on targeted tests was 45%. Thus, we estimate the number of mutation carriers detected April 1996–March 2020 to be 2129 (from full gene testing) and 5593 (from cascade testing). The predicted number of MMR mutation carriers in England is~200 000 (based on an estimated mutational prevalence of 1/279 and an estimated population size of 56 million).^3^ Thus, even allowing for variation in these estimates and activity subsequent to 2020, it is likely that we have identified fewer than 5% of the MMR mutation carriers in England. The modest number of individuals in whom targeted (predictive) germline MMR analysis has been performed is also noteworthy. While predicted yields from family cascading are often overestimated, these rates indicate that additional resource invested in familial cascading may be warranted following identification of familial probands.^24 25^

### Limitations

There are significant limitations to the NDRS germline MMR dataset, inherent in complete amalgamation under healthcare data governance rather than biased, patchy, incomplete opt-in via individual-level research consent. Due to changes in LIMS structures over time, laboratories were unable to extract and submit the totality of their historic NHS MMR analyses. The NDRS germline MMR dataset is only nationally complete for full-gene analyses from 2016 onwards. For 4 out of 13 laboratories, targeted gene analyses were not submitted. We were able to estimate gaps in the NDRS germline MMR dataset using CMGS/ACGS national audit data. However, these estimates are limited by the CMGS/ACGS audit data only dating from 1998 to 2016, including some non-NHS MMR and MSI analyses, and lacking breakdown into full gene versus targeted analyses.

The 13 laboratories that conducted NHS MMR analyses between 1996 and 2020 were provided with the NDRS common data model on which to design their local data extracts (online supplemental table 1). Some laboratories were able to submit structured data extracts with fields matching those in the common data model. However, many laboratories were not able to submit data in the structure requested, on account of limited local informatics/bioinformatics resource as well as the heterogeneity over time of their LIMS. For most laboratories, in order to reconstruct the data into the NDRS common data model, data items needed to be derived from multiple data fields or reference to external indices (eg, local laboratory test codes defining gene sets), or extracted from free text clinical report wording (eg, gene and variant data). Part of the laboratory-specific computational algorithms was recognition and extraction of HGVS-compliant variant nomenclature. Accordingly, the computational algorithms were not designed to recognise/extract informally or inaccurately described variants, which applied in particular to copy number variants. In the longer term, only through significant revision and consistency in the design of LIMS systems, as well as how they are populated from bioinformatics workflows, will it be possible to optimise accuracy and completeness of variant-level data for centralised submission and amalgamation in NDRS (or other central resources). In the meantime, manual review will be required to evaluate the 24% of abnormal results with non-recognisable variant nomenclature.

Missing patient identifiers for <1% of patient records prevented linkage to the NDRS cancer registry. However, for 8.6% of patient-level records, only pseudo-ID2 is available, for which linkage is less robust. In the NDRS germline MMR dataset, for patients who have undergone full-gene analysis and have sufficient pseudonyms available for linkage to the cancer registry, 70% linked to one or more registered cancer(s) diagnosed before their genetic test. Local audit in a subset of centres against locally held data on cancer diagnoses confirmed validity of scenarios in which there was non-linkage of full-gene analyses to registered cancers. Explanatory factors include MMR analyses being undertaken on account of benign tumours, family history of cancer and/or for syndromic features. However, some cancers were missed where the patient received cancer treatment outside of England or in the private sector (online supplemental methods). Pseudonymisation required on account of the healthcare data governance (rather than individual-level research consent), limited full investigation across the complete dataset for individuals not linking to the cancer registrations.

Prior to 2017, most individuals undergoing diagnostic testing for LS were preselected on the basis of meeting Amsterdam/Bethesda criteria defining ‘enrichment’ for personal or familial cancer history.^6 7^ This preselection continues currently for patients presenting to clinical genetics services with historic diagnoses or family histories of Lynch-related cancers. However, this runs in parallel to universal LS tumour screening of prospectively identified bowel and endometrial cancers.^8–10^ Case-specific clinical test indication and family history information was not collected in the NDRS germline MMR dataset. Hence, inability to deconvolute this mixed ascertainment limits some of the dataset’s uses for epidemiological and variant interpretation applications.

Due to Family IDs not being consistently held within laboratory LIMS systems, and lack of consistent national formats, it is not possible to link records for family members together. However, it has been standard practice to communicate across genetics centres to ensure full-gene testing is not undertaken in additional family members where a PV has already been ascertained in the family.

### Future directions

Additional analyses of the NDRS germline MMR dataset are underway to further evaluate the extracted variants, their nomenclature and pathogenicity according to current classification systems. This will provide insight into the accuracy by which variant nomenclature is ascribed by laboratories, the fidelity by which this can be extracted by computational algorithms from laboratory submissions, and more accurate estimation of the number of MMR PV carriers present within the dataset.

NICE guidance on universal LS tumour screening of bowel and endometrial cancers was published in 2017 and 2020, respectively. NDRS cancer registrations have included somatic molecular data since 2016 and MSI, MMR IHC, *BRAF* and *MLH1* methylation analysis is available for all tumours diagnosed from 2019 onwards. Together with linkage to national cancer registrations, this will allow comprehensive analysis of the full LS testing pathway, enabling evaluation of compliance with NICE guidance and equity across groups. Temporal evaluation of rates of full-gene NHS MMR analyses of probands, LS diagnoses and subsequent targeted analyses in relatives will be informative to evaluate the effectiveness of NICE recommendations.

Longitudinal outcome analysis of patients with LS and cancer, stratified by cancer treatment, will be possible via linkage to cancer registrations, HES, SACT, RTDS and ONS mortality data. Detailed pathology records are also available for all cancers diagnosed, with potential for identification of previously unrecognised features and subtypes. Longitudinal analysis of LS carriers without a previous diagnosis of cancer will enable study of cancer incidence and the impact of surveillance (eg, colonoscopy) and risk-reducing surgeries (eg, hysterectomy). Evaluation of the frequency of testing for and identification of Constitutional Mismatch Repair Deficiency (CMMRD) will be possible via analysis of probands undergoing MMR testing in childhood, in conjunction with instances of paediatric tumours and presence of dual mutations.

Furthermore, there is opportunity to use these data as the basis of Lynch syndrome patient registries, aimed at ensuring correct follow-up and management of mutation carriers as well as a resource for identifying individuals suitable for therapeutic trials. However, for a complete national registry, full retrieval of all MMR mutation carriers identified at each centre is required.

In regard of sustainability, infrastructure for submission of NHS germline genetic analyses data is now well-established and English laboratories conducting NHS MMR and other cancer susceptibility gene analyses submit regular data prospectively. This growing dataset will provide improved power for service evaluation and research. Furthermore, with migration in 2021 of the NDRS from PHE to NHS Digital/NHS England, the wider legal accountabilities for data capture provide additional momentum and reduce requirement for pseudonymisation.

### Summary

We have provided a description and analysis of amalgamated national germline MMR testing data from across English NHS regional molecular genomics laboratories from 1998 to 2020 with complete patient-level national data for full-gene analyses for 2016–2020. These data illustrate some of the opportunities, complexities and limitations inherent in national amalgamation of real-world genomic data from multiple laboratories, along with future directions by which the completeness and accuracy of this dataset will be improved. Collection of data under healthcare data governance rather than individual level research consent allows unbiased collection of complete national data, but adds challenges in regard of use of identifiers and data access. There is opportunity from this full national-level amalgamation of healthcare genomic data to generate a register of the patients analysed and a catalogue of the results detected. The location of these data within NDRS and NHS Digital allows prospective and retrospective linkage, not just to registered cancers, but also to national datasets holding patient characteristics such as ethnicity and geography, hospital episodes such as surgery and endoscopy and outcome data. Such national amalgamation of germline MMR testing data provides a unique opportunity for research, service evaluation and national patient registries and is, to our knowledge, the first of its kind worldwide.

## Data Availability

Data are available upon reasonable request. Data may be obtained from a third party and are not publicly available. All data relevant to the study are included in the article or uploaded as supplementary information. All summary data relevant to the study are included in the article or uploaded as online supplementary information. Individual level data detailed in this study are held within NHS Digital with access available on application.

## References

[1] Lynch HT , Snyder CL , Shaw TG , Heinen CD , Hitchins MP . Milestones of Lynch syndrome: 1895–2015. Nat Rev Cancer 2015;15:181–94. 10.1038/nrc3878 25673086

[2] Dominguez-Valentin M , Sampson JR , Seppälä TT , Ten Broeke SW , Plazzer J-P , Nakken S , Engel C , Aretz S , Jenkins MA , Sunde L , Bernstein I , Capella G , Balaguer F , Thomas H , Evans DG , Burn J , Greenblatt M , Hovig E , de Vos Tot Nederveen Cappel WH , Sijmons RH , Bertario L , Tibiletti MG , Cavestro GM , Lindblom A , Della Valle A , Lopez-Köstner F , Gluck N , Katz LH , Heinimann K , Vaccaro CA , Büttner R , Görgens H , Holinski-Feder E , Morak M , Holzapfel S , Hüneburg R , Knebel Doeberitz Mvon , Loeffler M , Rahner N , Schackert HK , Steinke-Lange V , Schmiegel W , Vangala D , Pylvänäinen K , Renkonen-Sinisalo L , Hopper JL , Win AK , Haile RW , Lindor NM , Gallinger S , Le Marchand L , Newcomb PA , Figueiredo JC , Thibodeau SN , Wadt K , Therkildsen C , Okkels H , Ketabi Z , Moreira L , Sánchez A , Serra-Burriel M , Pineda M , Navarro M , Blanco I , Green K , Lalloo F , Crosbie EJ , Hill J , Denton OG , Frayling IM , Rødland EA , Vasen H , Mints M , Neffa F , Esperon P , Alvarez K , Kariv R , Rosner G , Pinero TA , Gonzalez ML , Kalfayan P , Tjandra D , Winship IM , Macrae F , Möslein G , Mecklin J-P , Nielsen M , Møller P . Cancer risks by gene, age, and gender in 6350 carriers of pathogenic mismatch repair variants: findings from the prospective Lynch syndrome database. Genet Med 2020;22:15–25. 10.1038/s41436-019-0596-9 31337882 PMC7371626

[3] Win AK , Jenkins MA , Dowty JG , Antoniou AC , Lee A , Giles GG , Buchanan DD , Clendenning M , Rosty C , Ahnen DJ , Thibodeau SN , Casey G , Gallinger S , Le Marchand L , Haile RW , Potter JD , Zheng Y , Lindor NM , Newcomb PA , Hopper JL , MacInnis RJ . Prevalence and penetrance of major genes and polygenes for colorectal cancer. Cancer Epidemiol Biomarkers Prev 2017;26:404–12. 10.1158/1055-9965.EPI-16-0693 27799157 PMC5336409

[4] Yurgelun MB , Kulke MH , Fuchs CS , Allen BA , Uno H , Hornick JL , Ukaegbu CI , Brais LK , McNamara PG , Mayer RJ , Schrag D , Meyerhardt JA , Ng K , Kidd J , Singh N , Hartman A-R , Wenstrup RJ , Syngal S . Cancer susceptibility gene mutations in individuals with colorectal cancer. J Clin Oncol 2017;35:1086–95. 10.1200/JCO.2016.71.0012 28135145 PMC5455355

[5] Ryan NAJ , McMahon R , Tobi S , Snowsill T , Esquibel S , Wallace AJ , Bunstone S , Bowers N , Mosneag IE , Kitson SJ , O'Flynn H , Ramchander NC , Sivalingam VN , Frayling IM , Bolton J , McVey RJ , Evans DG , Crosbie EJ . The proportion of endometrial tumours associated with Lynch syndrome (petals): a prospective cross-sectional study. PLoS Med 2020;17:e1003263. 10.1371/journal.pmed.1003263 32941469 PMC7497985

[6] Vasen HF , Mecklin JP , Khan PM , Lynch HT . The International Collaborative group on hereditary non-polyposis colorectal cancer (ICG-HNPCC). Dis Colon Rectum 1991;34:424–5. 10.1007/BF02053699 2022152

[7] Rodriguez-Bigas MA , Boland CR , Hamilton SR , Henson DE , Jass JR , Khan PM , Lynch H , Perucho M , Smyrk T , Sobin L , Srivastava S . A national cancer Institute workshop on hereditary nonpolyposis colorectal cancer syndrome: meeting highlights and Bethesda guidelines. J Natl Cancer Inst 1997;89:1758–62. 10.1093/jnci/89.23.1758 9392616

[8] NHS-England . National genomic test directory, 2019. Available: https://www.england.nhs.uk/publication/national-genomic-test-directories/https://www.england.nhs.uk/publication/national-genomic-test-directories/

[9] NICE National Institute for Health and Care Excellence . Testing strategies for Lynch syndrome in people with endometrial cancer, 2020. Diagnostics guidance [DG42]

[10] National Institute for Health and Care Excellence . Molecular testing strategies for Lynch syndrome in people with colorectal cancer (DG 27), 2017. Available: https://www.nice.org.uk/guidance/dg27

[11] NHS England . Implementing Lynch syndrome testing and surveillance pathways, 2021.

[12] Henson KE , Elliss-Brookes L , Coupland VH , Payne E , Vernon S , Rous B , Rashbass J . Data resource profile: National cancer registration dataset in England. Int J Epidemiol 2020;49:16–16h. 10.1093/ije/dyz076 31120104 PMC7124503

[13] Stevens S , Miller N , Rashbass J . Development and progress of the National congenital anomaly and rare disease registration service. Arch Dis Child 2018;103:215–7. 10.1136/archdischild-2017-312833 29066522

[14] Public Health England . Systemic anti-cancer therapy dataset (SACT), 2018. Available: http://www.ncin.org.uk/collecting_and_using_data/data_collection/chemotherapy

[15] National Radiotherapy Dataset (RTDS) . Public Health England, 2018. Available: http://www.ncin.org.uk/collecting_and_using_data/rtds

[16] NHS Digital . Hospital episode statistics, 2018. Available: http://content.digital.nhs.uk/hes

[17] Knoppers BM . International ethics harmonization and the global alliance for genomics and health. Genome Med 2014;6:13. 10.1186/gm530 25031613 PMC3979077

[18] Digital N . Appendix 1: section 251 of the National health service act 2006; 2019.

[19] Legislation.gov.uk . Section 254 of the health and social care act; 2012.

[20] Public Health England . Accessing PHE data through the office for data release; 2021.

[21] Public Health England . Independent Advisory panel on data release; 2021.

[22] NHS Digital . Transition of arrangements for governance of data access from the UKHSA office for data release (ODR) to the NHS digital data access request service (DARS); 2022.

[23] Richards S , Aziz N , Bale S , Bick D , Das S , Gastier-Foster J , Grody WW , Hegde M , Lyon E , Spector E , Voelkerding K , Rehm HL , ACMG Laboratory Quality Assurance Committee . Standards and guidelines for the interpretation of sequence variants: a joint consensus recommendation of the American College of medical genetics and genomics and the association for molecular pathology. Genet Med 2015;17:405–24. 10.1038/gim.2015.30 25741868 PMC4544753

[24] Pashayan N , Turnbull C . Peridiagnostic and cascade cancer genetic testing. Nat Rev Clin Oncol 2020;17:277–8. 10.1038/s41571-020-0348-4 32152486

[25] Woodward ER , Green K , Burghel GJ , Bulman M , Clancy T , Lalloo F , Schlecht H , Wallace AJ , Evans DG . 30 year experience of index case identification and outcomes of cascade testing in high-risk breast and colorectal cancer predisposition genes. Eur J Hum Genet 2022;30:413–9. 10.1038/s41431-021-01011-8 34866136 PMC8645350

