# Supplementary material for: Germline mismatch repair (MMR) gene analyses from English NHS regional molecular genomics laboratories 1996–2020: development of a national resource of patient-level genomics laboratory records
Source: J Med Genet. 2022 Dec 26;60(7):669–78. doi: 10.1136/jmg-2022-108800 (PMC10359571; doi:10.1136/jmg-2022-108800)
Supplement: Supplementary data [file jmg-2022-108800supp001.pdf]

Germline Mismatch Repair (MMR) gene analyses from English NHS regional molecular genetics laboratories 1996-2020: development of a national resource of patient-level laboratory data

L. Loong[1]*, C. Huntley[1]*, F. McRonald[2]*, F. Santaniello[2, 3]*, J. Pethick[2], B. Torr[1], S. Allen[1], O.Tulloch[2, 3], S. Goel[2, 3], B. Shand[2, 3], T. Rahman[2, 3], M. Lüchtenborg[2, 4], A. Garrett[1], R. Barber[5], T. Bedenham[6], D. Bourn[7], K. Bradshaw[8], C. Brooks[9], J. Bruty[10], G. Burghel[11], S. Butler[5], C. Buxton[12], A. Callaway[13], J. Callaway[13], J. Drummond[10], M. Durkie[14], J. Field[8], L. Jenkins[9], T. P. McVeigh[1, 15] R. Mountford[16], R. Nyanhete[14], E. Petrides[6], R. Robinson[17], T. Scott[17], V. Stinton[16], J. Tellez[7], A. Wallace[11], L. Yarram-Smith[12], K. Sahan[18], N. Hallowell[18], D. Eccles[19,20], P. Pharoah[21], M. Tischkowitz[21], A. C. Antoniou[21], DG. Evans[11,22], F. Lalloo[11], G. Norbury[23], E.J.A. Morris[24] J. Burn[25] †, S.A. Hardy[2] †, C. Turnbull[1, 15] †

Supplementary Methods

Table of Contents

NDRS Optimisation and quality assurance of laboratory-specific data extraction and restructuring algorithms *3*

Structure of the NDRS germline MMR dataset *3*

Analysis and Data Linkage *4*

Defining a germline MMR gene analysis 4

Defining a unique patient 4

Defining a test episode 4

Imputation of total historic national laboratory activity (Figure 1, Supplementary figure 1, Supplementary table 2) 5

Cancer registrations (Figure 3 and Supplementary Table 3) 6

Mutalyzer 2.0.35 HGVS variant nomenclature check 6

NDRS Validation of Linkage to the Cancer Registry: *7*

NDRS Optimisation and quality assurance of laboratory-specific data extraction and restructuring algorithms

- A laboratory-specific set of restructuring rules were developed by NDRS in communication with the laboratory, to specify the data mapping, data item derivations and regular expression recognition required to populate the NDRS common data model from the laboratory data extract.
- Rules are converted into Ruby (programming language) and a first trial extraction and restructuring conducted. Gene-specific counts of total full gene analyses, abnormal full gene analyses, total targeted analyses and abnormal targeted analyses were generated. These counts were compared with the original laboratory data extract.
- Discrepancies were examined, code refined, and counts compared iteratively until there was <5% difference between the extracted restructured data and visual inspection of original laboratory data extracts.
- Test suites were written.
- The Ruby code was refactored to comply with NHSD and professional standards and published on GitHub as an official record of the data processing.
- The imported data is copied into the NDRS Cancer Analysis System (CAS) (an SQL relational database) where it can be accessed and linked to additional NDRS datasets including the national cancer registry.
- A final comparison of total full gene analyses, abnormal full gene analyses, total targeted analyses, abnormal targeted analyses and specific variant counts was made between the original laboratory data extract, and the restructured data pre- and post- upload to the CAS.

Structure of the NDRS germline MMR dataset

The NDRS germline MMR dataset is a SQL relational database. The data items are stored in a hierarchical data model of 4 linked tables arranged sequentially in a one-to-many relationship. The four tables hold (i) pseudonymised patient-identifiers, (ii) test episode data, (iii) per-gene results data, (iv) sequence variants (Figure a).

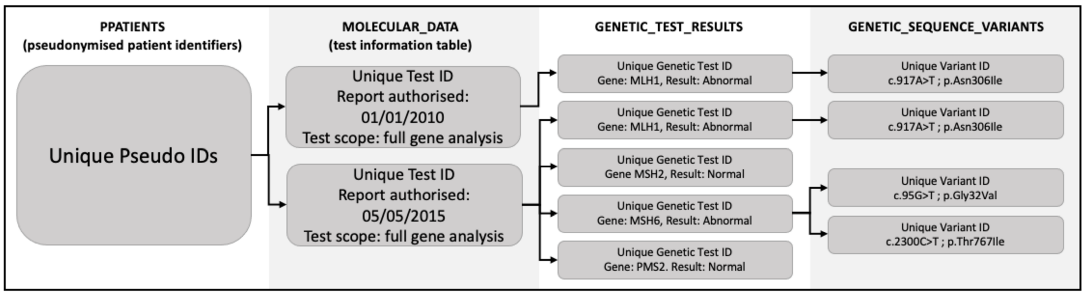

Figure a. Schematic showing the hierarchical data model and one-to-many relationship between the constituent tables of the NDRS germline MMR dataset. In the example a single individual had two temporally distinct testing episodes in 2010 and 2015. In the first, single gene testing of *MLH1* identified one variant. In the second testing episode, all 4 MMR genes were tested, two variants in *MSH6* were identified in addition to the original *MLH1* variant which was again identified.

Analysis and Data Linkage

Defining a germline MMR gene analysis

For all analyses in this study, a germline MMR gene analysis was defined by two criteria:

1. Test indication as stated by the submitting laboratory was a full-gene or targeted analysis undertaken for a personal and/or family history of bowel or Lynch-related cancer and/or clinical suspicion for Lynch syndrome.
   -AND-
2. The test episode includes analysis of at least one gene out of MLH1, MSH2, MSH6, PMS2, EPCAM.

Defining a unique patient

A single patient may have been seen and received genetic testing from a clinical genetics service more than once. There are also duplicate patient-level records in the NDRS germline MMR dataset where patient-level records have been included in laboratory data extracts more than once.

For all analyses a unique patient is defined hierarchically as follows:

1. a distinct Pseudo-ID1 (created from the NHS number), or if that is unavailable
2. a distinct Pseudo-ID2 (created from the DoB and postcode), or if that is unavailable
3. a distinct local laboratory identifier.

Defining a test episode

Several laboratories submitted patient-level records with different test report authorisation dates for different elements of the same MMR gene analysis. For example, if an MLPA run for copy number variants is conducted on a different day to an NGS run for sequence variants, or if PMS2 is analysed on a different day to MLH1/MSH2/MSH6, then these records could have different report authorisation dates. Regarding these analyses as a single test episode was necessary to ensure that estimates of the total number of MMR analyses were not inflated by test episodes reported over several dates, and to ensure that where dates fall across a year divide they are not counted in both years.

For patients with more than one test report authorisation date, the earliest date is taken as the test-episode date. All subsequent records with test report authorisation dates within 365 days of the test-episode date, have their test report authorisation dates reassigned as the test-episode date. This process is repeated three times. (Figure b.)

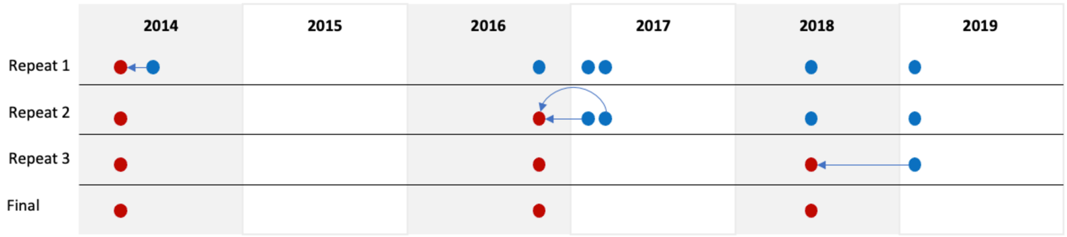

Figure b. Dots represent test authorisation dates. In each repeat, the earliest test authorisation date (red dot) is assigned as the test episode date. All test authorisation dates associated with the same

unique person that fall within 365 days of the test episode date are reassigned to the test episode date (arrows). In the example a unique person with seven separate test authorisation dates is rationalised to three temporally distinct test episodes.

Number of test episodes	Number of patients
1	16,137
2	406
3	30
>3	3

Table a. Number of test episodes assigned to each unique patient in the NDRS germline MMR dataset.

Imputation of total historic national laboratory activity (Figure 1, Supplementary figure 1, Supplementary table 2)

In order to estimate overall numbers of NHS MMR analyses conducted since the initiation of this testing in 1996 and the proportion that are captured in the NDRS germline MMR dataset, it was necessary to estimate the number of NHS MMR analyses (total, full-gene, and targeted analyses) undertaken at each laboratory historically.

Data were retrieved from the Clinical Molecular Genetics Society/Association of Clinical Genomic Science (CMGS/ACGS) annual per-laboratory audit of MMR analyses, which covered financial years 1998-2016. These counts included all English NHS MMR analyses (full-gene and targeted) performed by each laboratory in a financial year, but for some laboratories were inflated by inclusion of tests for other patients (devolved nations, overseas, private, research) and MSI analyses.

The following adjustments were made (Supplementary Table 2):

(i) **Activity 1996-7 and 1997-8.** First NHS MMR analyses were reported in 1996, but the CMGS/ACGS audit was only initiated in 1998. Activity for these two years was estimated by ascribing for these two years the same laboratory-specific activity registered for 1998-9.

(ii) **The proportion of MMR analyses in the CMGS/ACGS data which comprised germline MMR analyses specific to English NHS patients** (versus devolved nations, overseas, private and research patients and MSI analyses). This was estimated by comparing analyses counts in the CMGS/ACGS audit to counts in the NDRS dataset, for years where both were available, to generate a laboratory-specific adjustment factor (sum of CMGS/ACGS audit analyses over the sum of NDRS total analyses for the overlapping time-period. This adjustment was then applied to 'raw' CMGS/ACGS analyses counts for the years pre-dating NDRS data submissions, to generate 'down-adjusted' CMGS/ACGS MMR analyses counts approximating germline MMR analyses specific to English NHS patients.

(iii) **Targeted MMR analyses counts for the 4/13 laboratories not submitting all targeted analyses to NDRS**. A year-specific full-gene analyses to targeted analyses ratio was generated using counts of full-gene and targeted analyses submitted to NDRS by the other

9/13 laboratories. This ratio was applied to the full-gene counts for the 4 laboratories to estimate their targeted analyses counts.

(iv) **Breakdown of full gene versus targeted MMR analyses for years pre-dating NDRS submission (for which only CMGS/ACGS audit data were available).** This was estimated by applying the year-specific full-gene analyses: targeted analyses ratio calculated above, to the 'down-adjusted' CMGS/ACGS MMR analyses counts. For years where a year-specific full-gene analyses: targeted analyses ratio was incalculable, the average ratio for the calculable years was applied (1.93).

(v) **Estimate counts of total, full-gene and targeted NHS MMR analyses for the entire period between April 1996 - March 2020**. These were derived from integration of counts of NHS MMR analyses in the NDRS germline MMR dataset where these were available and complete for a financial year. For years pre-dating NDRS data submission, the 'down-adjusted' counts derived from CMGS/ACGS audit data were used.

Both NDRS and CMGS/ACGS counts include a small number of repeat MMR gene analyses for returning patients receiving subsequent MMR gene analyses from clinical genetics after ≥ 1 year (See above – defining a test episode). Patients in the NDRS germline MMR dataset with >1 test episode = 439.

Cancer registrations (Figure 3 and Supplementary Table 3)

Linkage to the National Cancer Registration and Analysis Service (NCRAS) national cancer registry was performed using pseudo-ID1 and pseudo-ID2 separately. Where linkage was successful, 3-character ICD10 site codes were retrieved from the AV2019 tumour table (national cancer registrations up to the end of 2019). Retrieved cancer registrations were filtered as per NCRAS internal case counting standard operating procedures to remove: non-finalised cancer registrations, duplicate cancer registrations, cancers diagnosed before 1995, non-malignant neoplasms and non-melanoma skin cancers. Cancer registrations that successfully linked twice using both pseudo-IDs were deduplicated.

For figure 3 and supplementary table 3 unique cancer registrations are counted. For unique patients in the NDRS germline MMR dataset that linked to a cancer registration, whether that cancer was diagnosed before or after their germline MMR analysis was determined relative to their first test episode (if they had had multiple). Multiple primary cancer registrations in the same patient are counted in figure 3 and supplementary table 3. Cancers diagnosed before and after a MMR analysis are also both counted.

When calculating proportions of patients that link to a registered cancer, patients without linkage IDs (pseudo-ID1 and pseudo-ID2) are first excluded.

Mutalyzer 2.0.35 HGVS variant nomenclature check

Variants extracted from abnormal results in the NDRS germline MMR dataset were concatenated with NCBI reference sequences (MLH1 NM_000249.3; MSH2 NM_000251.2; MSH6 NM_000179.2; PMS2 NM_000535.5; EPCAM NM_002354.3) and run through the Mutalyzer 2.0.35 Batch name checker. Intronic and untranslated region variants were converted to their corresponding chromosomal variant names using the Mutalyzer position converter and then put through the batch name checker. Of 4107

patient variants 3946 (96%) passed the HGVS check without any errors or warnings, and 161 (4%) failed.

NDRS Validation of Linkage to the Cancer Registry:

Purpose:

To evaluate integrity of the pseudonym matching process between the NDRS germline MMR dataset and other NDRS datasets including the NCRAS national cancer registry.

Method 1: External registries

Two datasets of patients with confirmed Lynch syndrome containing patient identifiers and cancer diagnoses were sourced for use as validation datasets (Newcastle University CAPP3 clinical trial and St. Mark's Hospital Polyposis registry).

Pseudo-ID1 and Pseudo-ID2 were generated for the two datasets using the same algorithms and application programming interface used for laboratory submission of data extracts to NDRS. Linkage of the two datasets using the Pseudo-IDs to the national cancer registry was undertaken. Where a patient flagged in the validation dataset as having cancer did not link to a cancer registration, a manual check of the cancer registry was undertaken using unencrypted patient identifiers.

Method 2: Laboratory test indication audit

As it was anticipated that most full gene germline analyses would be conducted in probands with cancer, each regional molecular genetics laboratory was asked to conduct an audit of up to 20 cases, supplied by NDRS which:

(1) were full gene analyses of MMR/CRC gene panel
(2) with a valid Pseudo-ID1 (generated from NHS number)
(3) and yet did not link to a cancer registration.

Laboratories were requested for each case to provide the clinical test indication including cancer status. Where this indication included a personal history of cancer, unencrypted patient identifiers were requested to be checked against the cancer registry.

Results 1: External registries

There were 812 individuals with confirmed Lynch syndrome provided by the two external registry datasets, of whom 413 were reported to have a cancer diagnosis. 346/413 (84%) patients reported to have cancer matched a cancer registration in the national cancer registry. Of the 67/413 (16%) patients reported in the external registry datasets to have cancer but not linking to cancer registration, 37 (i) had missing or incorrect NHS numbers/ dates of birth/ postcodes such that valid Pseudo-IDs had not been generated for them, or (ii) were resident outside of England or Wales. 30 patients were reported as having cancer in the external registries, were resident in England/Wales, had appropriate Pseudo-IDs but lacked cancer registrations on the national cancer registry (Figure c.)

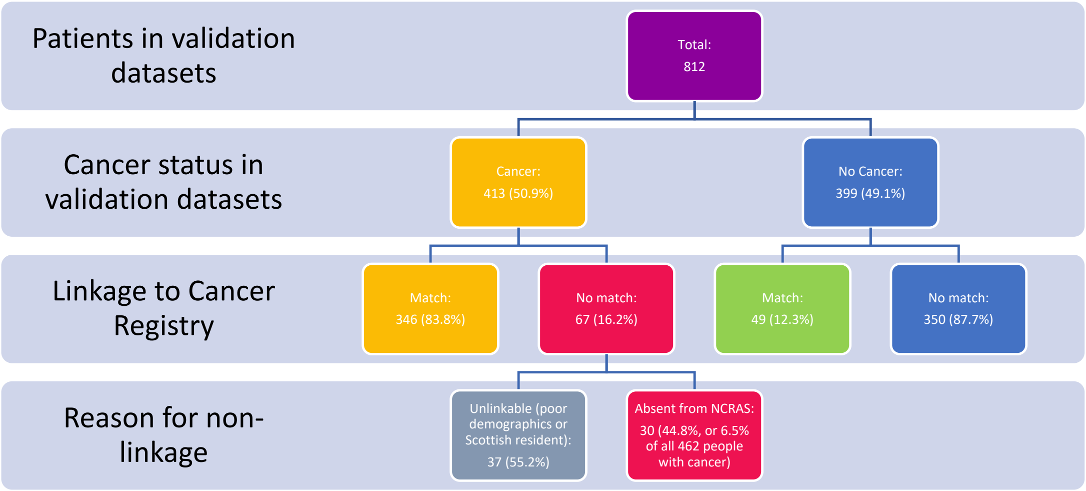

Figure c. Schematic of validation process

Results 2: Laboratory test indication audit

For >70% of patients in whom CRC/MMR full gene analysis was performed there was successful link to a cancer registration pre-dating the CRC/MMR analysis in the national cancer registry. In this audit, we collected data on clinical test indication for a subset of the 30% of patients receiving full gene analyses who did not link to a registered cancer. 10/12 laboratories asked to participate in the audit responded, encompassing 189 cases. Results are shown in the table below. 77% of the cases audited did not have cancer but had been offered full gene analysis on the basis of benign tumours, family history or syndromic features. For 24/189 (12.7%) the laboratory reported a cancer being documents on their LIMS system at time of MMR testing, but the cancer could not be identified on the cancer registry.

Reason for no match to Cancer Registry	Number (Percentage)
Unaffected screen (benign tumours, +/- family history +/- self-pay)	132 (69.8%)
Clinically diagnosed or suspected syndromic features (PJS manifestations / macrocephaly + developmental delay [PTEN] / CHRPE etc.)	14 (7.4%)
Clinical history unknown (but lab has no info to suggest cancer)	9 (4.8%)
Targeted test on unaffected person (i.e. scope miscoded by lab)	2 (1.1%)
Carrier screening for MAP (MUTYH variants)	4 (2.1%)
Demographic discrepancies affecting both pseudoIDs	2 (1.1%)
Not resident in England	2 (1.1%)
Patient (or tumours) cannot be found on cancer registry	24 (12.7%)
**TOTAL**	**189 (100%)**

Table b. Summary of reasons for no match to cancer registry

Further investigations:

The 30 cases in the external registries and the 24 cases from the laboratory audits (54 total) who were reported to have cancer but did not link to a registered cancer in the national cancer registry, were further investigated using remote access to hospital record systems available to Cancer Registration Officers within the NCRAS. Due to COVID-19-related restrictions to hospital data, so only 30/54 records

could be checked. Of those 30 cases checked, the outcomes are shown in the table below. 10/30 were benign tumours, 10/30 were non-English residents or private patients whose cancers would not be registered in the English national cancer registry, and for 6/30 no evidence of cancer was found on the trust system. For the remaining four cases, one was a true invasive cancer diagnosed in 1972, one was a very recent diagnosis not yet captured on CAS and for two there was indirect mention of the cancer in the clinical notes but no formal coding of invasive cancer for the patient

**Outcome of checks**	**Count of cases**
No access to Trust system	24
Tumour missing from cancer registry: (tumour from 1972)	1
Recent diagnosis; not yet on cancer registry	1
Cancer history mentioned indirectly in clinical notes –cancer not directly coded I patient record	2
No evidence for tumour found on Trust system	6
Private patient	4
Patient likely diagnosed / treated in Scotland or overseas	6
Benign / non-registrable tumour(s) only	10
**TOTAL**	**54**

Table c. Summary of outcomes of checks

Conclusion:

In summary, the sequential audit processes provide robust assurances regarding NDRS creation of linkage IDs (pseudo-ID1 and pseudo-ID2), the process of linkage to the national cancer registry, and registration of cancers. The majority of full gene analyses for which there is no linkage to the cancer registry are explained by (i) incomplete NHS numbers/ dates of birth/ postcodes which preclude the generation of linkage pseudo-IDs, (ii) patients receiving full-gene analyses for reasons other than a personal history of invasive cancer, including benign tumours (iv) cancer diagnosis outside of England or in the private sector.
