# Supplementary material for: Germline mismatch repair (MMR) gene analyses from English NHS regional molecular genomics laboratories 1996–2020: development of a national resource of patient-level genomics laboratory records
Source: J Med Genet. 2022 Dec 26;60(7):669–78. doi: 10.1136/jmg-2022-108800 (PMC10359571; doi:10.1136/jmg-2022-108800)
Supplement: Supplementary data [file jmg-2022-108800supp002.pdf]

Germline Mismatch Repair (MMR) gene analyses from English NHS regional molecular genetics laboratories 1996-2020: development of a national resource of patient-level laboratory data

L. Loong[1]*, C. Huntley[1]*, F. McRonald[2]*, F. Santaniello[2, 3]*, J. Pethick[2], B. Torr[1], S. Allen[1], O.Tulloch[2, 3], S. Goel[2, 3], B. Shand[2, 3], T. Rahman[2, 3], M. Lüchtenborg[2, 4], A. Garrett[1], R. Barber[5], T. Bedenham[6], D. Bourn[7], K. Bradshaw[8], C. Brooks[9], J. Bruty[10], G. Burghel[11], S. Butler[5], C. Buxton[12], A. Callaway[13], J. Callaway[13], J. Drummond[10], M. Durkie[14], J. Field[8], L. Jenkins[9], T. P. McVeigh[1, 15] R. Mountford[16], R. Nyanhete[14], E. Petrides[6], R. Robinson[17], T. Scott[17], V. Stinton[16], J. Tellez[7], A. Wallace[11], L. Yarram-Smith[12], K. Sahan[18], N. Hallowell[18], D. Eccles[19,20], P. Pharoah[21], M. Tischkowitz[21], A. C. Antoniou[21], DG. Evans[11,22], F. Lalloo[11], G. Norbury[23], E.J.A. Morris[24] J. Burn[25] †, S.A. Hardy[2] †, C. Turnbull[1, 15] †

Supplementary Figures

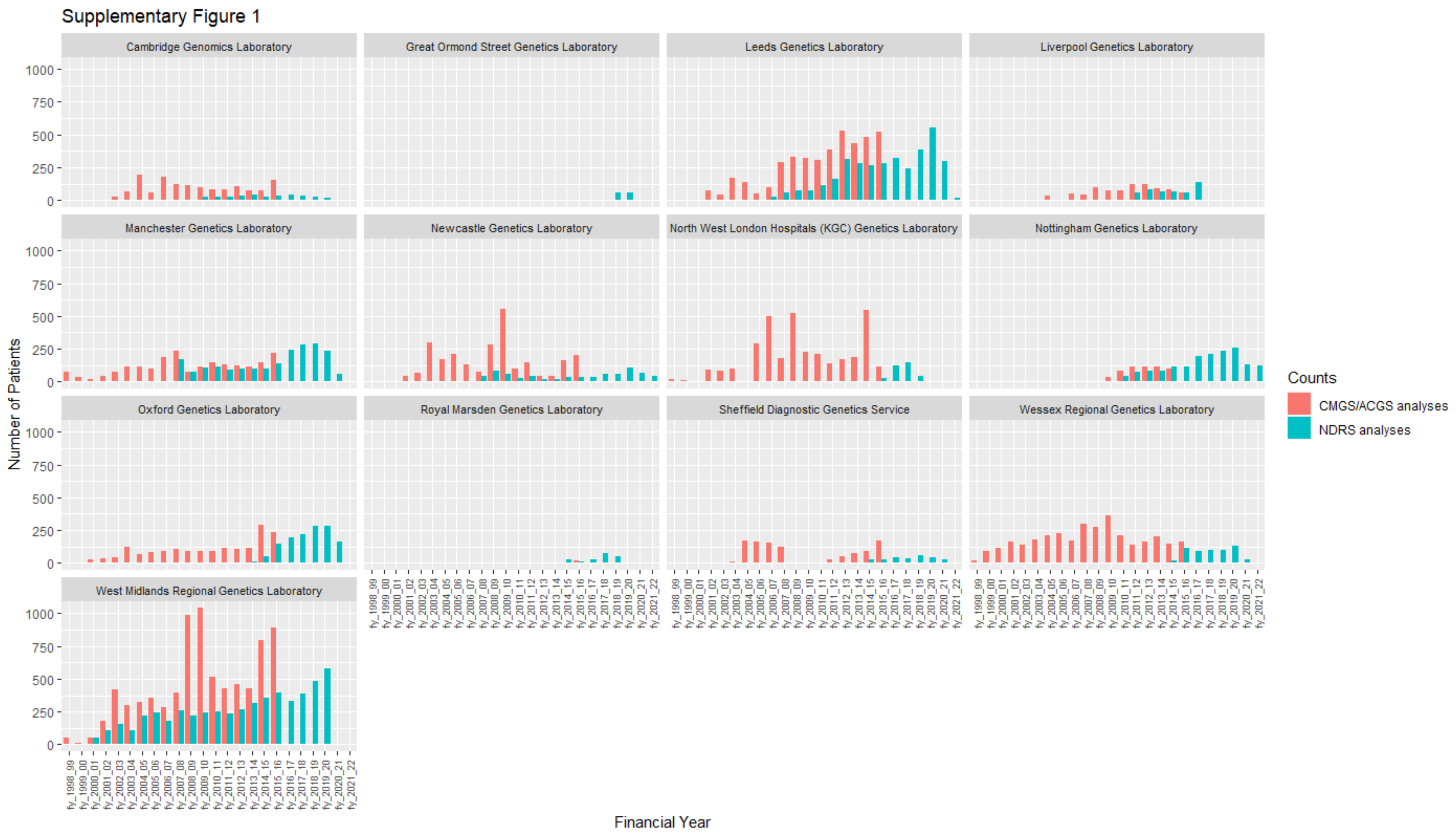

Supplementary Figure 1. Comparison of counts of NHS germline MMR gene analyses in the NDRS germline MMR dataset and in CMGS/ACGS annual MMR testing activity audit data, by laboratory. X-axis: Financial years Apr 1998 – Mar 2022. **Y-axis:** Number of patients tested in a financial year. **Turquoise:** Counts of patients who have received NHS MMR analyses and are captured in the NDRS MMR dataset. **Orange:** MMR analyses counts in the CMGS/ACGS audit data. The CMGS/ACGS counts include all full-gene and targeted English NHS germline MMR analyses, but also include NHS patients in devolved nations, overseas, private, and research patients, and MSI analyses. No laboratory was able to extract the entirety of their historic MMR analysis data for submission to NDRS and the earliest years of data submitted to NDRS varied from 2000 to 2015. Both NDRS and CMGS/ACGS data included a small number of repeat MMR analyses for patients returning to clinical genetics services and receiving subsequent MMR gene analyses. Patients in the NDRS germline MMR dataset with >1 test episode = 439 (See Supplementary Methods).

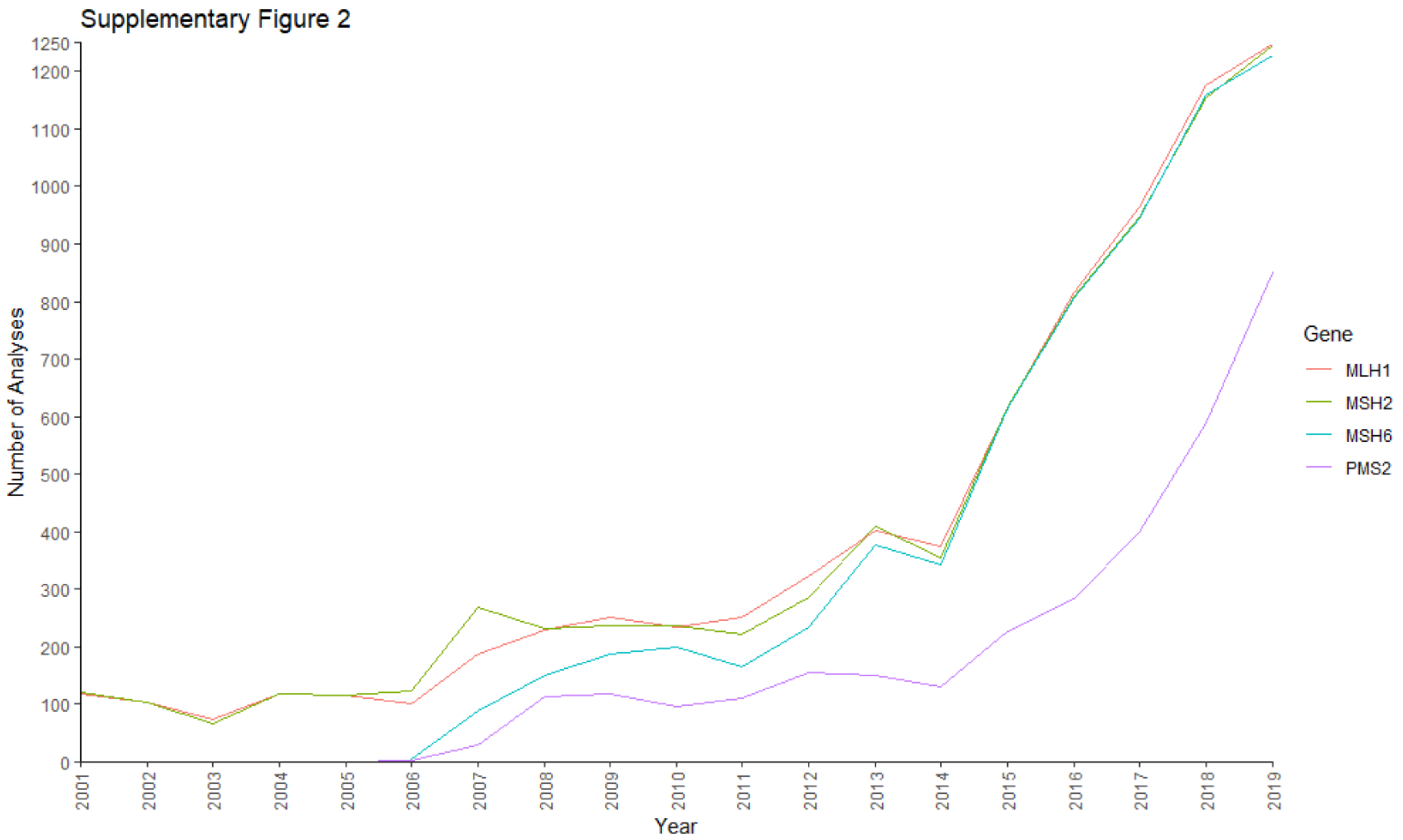

Supplementary Figure 2. Number of patients receiving full gene germline MMR gene analyses of each MMR gene by year. For the small number of patients with multiple test episodes over different years, these analyses are included in the counts. Prior to 2017 the counts reflect activity in a subset of laboratories so national volumes of analyses for each gene can not be inferred from this figure.

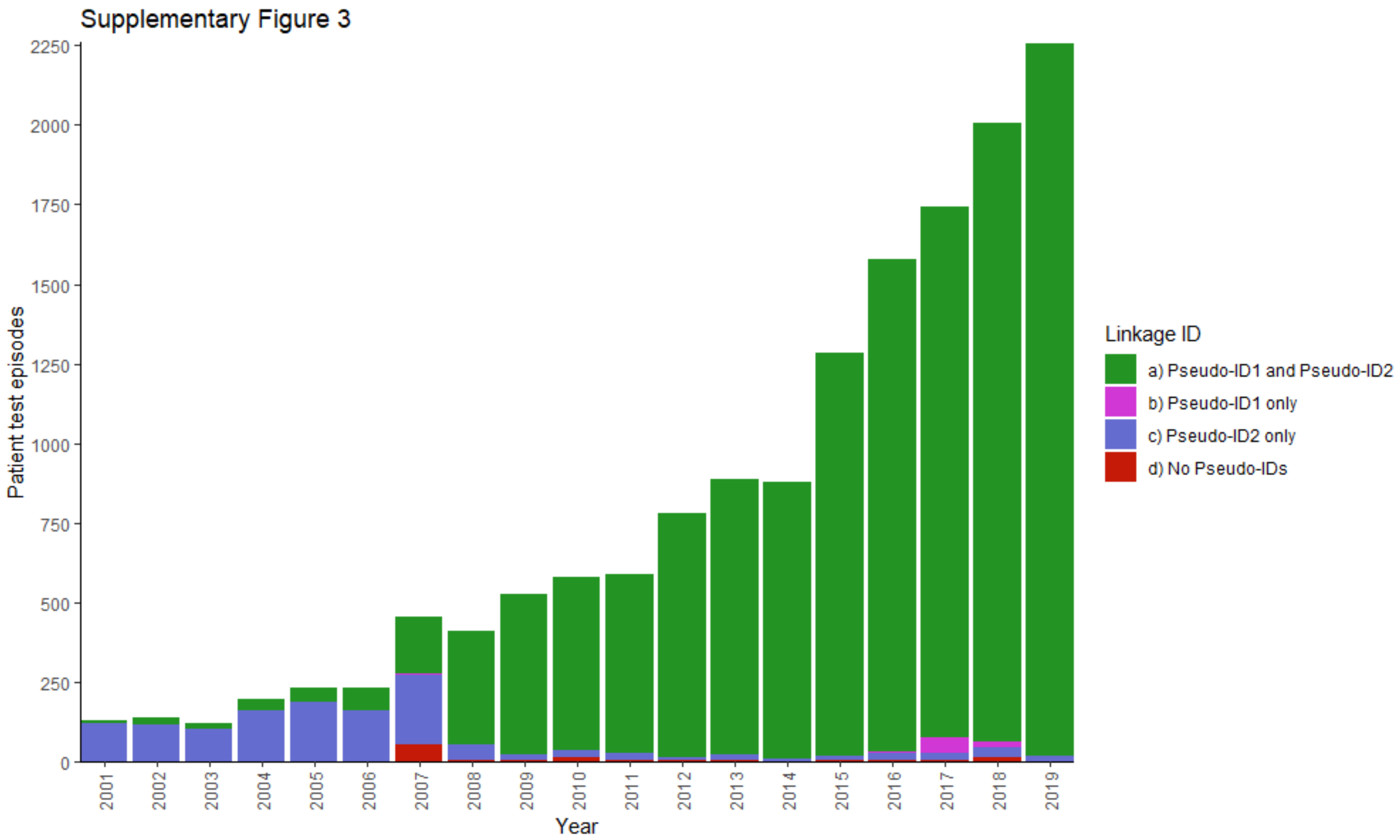

Supplementary Figure 3. Availability of pseudo-IDs to facilitate linkage to cancer registrations by year. X-axis: Calendar years. Y-axis: Patient test episodes. (For patients with multiple test episodes, all test episodes are included in their respective years).
